# Randomized controlled trial of immediate alveolar ridge preservation for bone and soft tissue aesthetics after tooth extraction

**DOI:** 10.3389/fsurg.2026.1771200

**Published:** 2026-03-12

**Authors:** Yi-Ye Zhou, Nan Li, Yan Gao, Hui Chen

**Affiliations:** Department of Stomatology, JinShan Hospital, Fudan University, Shanghai, China

**Keywords:** after tooth extraction, alveolar bone resorption, alveolar ridge preservation, collagen barrier membrane, randomized controlled trial, soft tissue aesthetics, *β*-tricalciumphosphate (*β*-TCP)

## Abstract

**Objective:**

This study aimed to evaluate the long-term outcomes of alveolar ridge preservation (ARP) on soft tissue morphology, correlate them with underlying bone changes, and assess its clinical value. It directly compared alveolar bone preservation and soft tissue aesthetics between ARP and conventional tooth extraction.

**Methods:**

In this prospective randomized controlled trial, sixty patients were randomly assigned (1:1) via sealed envelopes to a control group (conventional extraction) or an ARP group (immediate grafting with *β*-TCP and a collagen membrane). Over a 12-month follow-up, alveolar bone height and width changes were measured using CBCT. Soft tissue morphology was examined via plaster models, and aesthetics were evaluated using a 10-point scoring system for color and morphology.

**Results:**

Baseline characteristics were comparable between groups. After 12 months, the control group showed significantly greater bone resorption in height (1.3 mm) and width (1.7 mm) compared to the ARP group (0.7 mm and 1.1 mm, respectively; *P* < 0.001). Soft tissue contours were significantly better in the ARP group (50.0% smooth, 50.0% mild abnormalities) than in the control group (33.3% margin disruption, 60.0% moderate irregularities; *P* < 0.001). A strong negative correlation was found between aesthetic scores and bone resorption (*P* < 0.001). The ARP group also achieved significantly higher soft tissue color (7.8 ± 0.5) and morphological scores (7.7 ± 0.6) than the control group (6.1 ± 0.6 and 5.9 ± 0.7; *P* < 0.001).

**Conclusions:**

Immediate ARP using *β*-TCP and a collagen membrane significantly reduces alveolar bone resorption, better maintains soft tissue morphology and aesthetics, and provides a superior foundation for future implant rehabilitation, demonstrating substantial clinical value.

## 1 Introduction

The occlusal stimulation and occlusal remodelling of periodontal complex is lost following extraction of the tooth which leads to alveolar bone resorption. This important physiological process contributes to the issues of implant rehabilitation and aesthetic deficiency (1, 2). Research in histology has revealed a series of events that takes place in untreated extraction sockets: filling of the socket with granulation tissue within 8 weeks, formation of woven bone, and formation of lamellar bone maturation after lamellar bone maturation. However, in the presence of such a period, there is irreversible atrophy of the ridge, an average decrease in the height of from 1.0–2.0 mm and width of not less than 30%–50% in 12 months, especially on the frontal parts (3, 4). Restorative procedures, including implant procedure or fixed bridge reconstruction, are more complex due to gingival recession, deformities of the contours and decreased keratinized mucosa, which are due to morphological changes of both hard and soft tissues (5, 6).

One important tactic to reduce bone loss and keep soft tissues intact are alveolar ridge preservation (ARP) procedures that involve biomaterial grafting, as well as barrier membrane covering (7). An osteogenic milieu may be provided by such mechanisms as the “cell exclusion” function of barrier membranes and the “space-making” action of biomaterials and they underlie the concept of guided bone regeneration (GBR) (8). While previous research has shown that ARP can reduce alveolar height loss by 40%–60% compared to traditional extraction methods, most of these studies have been conducted on radiographic assessment methods for bone volume (e.g., height/width from CBCT), rather than assessing multidimensional correlations between bone support and soft tissue aesthetic dynamic changes (e.g., gingival margin arc, colour uniformity) (9, 10). Even in the event that bone volume is maintained, inferior clinical results may be related to neglecting soft tissue dynamics in the case of front teeth, which are of greatest importance aesthetically (11).

The purpose of this study is to benchmark the esthetic results of alveolar ridge preservation (ARP) compared to the conventional extraction using 3 supplementary assessment methods: radiographic bone collapse analysis for hard tissue support, soft tissue aesthetic scoring for colour and texture and plaster model morphology evaluation for 3D soft tissue contour. It goes into 3 reasonably large areas: (1) the long term (to 12 months) effects of ARP on the morphology of soft tissues, (2) the relationship between the aesthetics of soft tissues to the height/width of bone support structures and (3) how ARP will practically be used in individualized treatment plans (front vs. back teeth, different bone densities, etc). The results will validate the process by which the support of bone volume influences the aesthetics of soft tissues, and specifically, it will be possible to keep the three-dimensional structure of bone, which keeps the anchorage sites and vascular networks of the soft tissues intact, delaying the recession of the gums (12, 13), and will provide evidence-based knowledge for optimizing ARP applications (such as selection of biomaterial and type of membrane).

## 2 Methods

### 2.1 Study population and randomization

This prospective randomized controlled trial was designed in accordance with the CONSORT 2025 Statement for Randomized Controlled Trials. Initiated in 2022, the study was not registered at [ClinicalTrials.gov] as international clinical trial registration was not a mandatory institutional requirement for clinical research at the time of study design and implementation. All study procedures were approved by the ethics committee and conducted in full compliance with the Declaration of Helsinki.

Inclusion criteria: (1) Patients between the ages of 18 and 60 (to rule out conditions related to bone metabolism that develop with age); (2) reason to remove a single tooth [remaining crown or root out of trauma or caries, without severe periodontal disease (probing depth <6 mm and clinical attachment loss <4 mm)]; (3) The preoperative CBCT should show a height and width of a minimum of 5 mm in the alveolar bones to provide a baseline volume measurement that can be used for measurement purposes.; (4) Willingness to maintain optimal oral hygiene and complete the 12-month follow-up sessions; (5) Extraction sockets classified as Type I (intact buccal/labial cortical plate with 4 intact socket walls) based on the cortical plate integrity and residual socket wall number (14, 15).

Exclusion criteria: (1) Severe periodontitis (probing depth of 6 mm or more and a clinical loss of attachment of 4 or more); (2) Diseases such as pericoronitis and acute apical periodontitis may develop after the extraction at the extraction site.; (3) History of heavy smoking (ten cigarettes or more every day for at least five years), smoking will reduce the ability of the body to make new bone (16).; (4) chronic disorders (e.g., osteoporosis, cardiovascular disease with the need for anticoagulants, uncontrolled diabetes with a hemoglobin A1c level of 8% or higher); (5) Long-term use of drugs that alter metabolic system of bone (glucocorticoids, bisphosphonates); (6) pregnancy or lactation; (7) Extraction sockets with buccal/labial cortical plate defects (Type II/III) or less than 3 residual socket walls (14, 15).

Socket classification criteria: The extraction sockets were classified according to the cortical plate integrity and residual socket wall number (14, 15), a widely used classification system for alveolar ridge preservation studies:

Type I: Intact buccal/labial and lingual/palatal cortical plates, with 4 intact residual socket walls (mesial, distal, buccal/labial, lingual/palatal); no bony defects or fenestrations at the extraction site.

Type II: Partial buccal/labial cortical plate defect (≤1/3 of the plate height) with 3 intact residual socket walls; no obvious bone loss at the alveolar crest.

Type III: Severe buccal/labial cortical plate defect (>1/3 of the plate height) with ≤2 intact residual socket walls; accompanied by alveolar crest bone loss.

Only Type I extraction sockets were included in this study to eliminate the confounding effect of pre-existing bony defects on post-extraction alveolar bone resorption and soft tissue remodeling.

Between January 2022 and December 2022, the patients were recruited from the Department of Oral and Maxillofacial Surgery of Jinshan Hospital, Fudan University. Eight people were excluded from the final report after screening: three with periodontal disease, 2 with diabetes, 2 smokers and 1 person who did not return for follow-up prior to randomization. The other 60 patients were enrolled in the study and randomly assigned to the control group (*n* = 30) or ARP group (*n* = 30) by using the statistician-generated random number table.

Randomization and allocation concealment: A statistician independent of the clinical team generated a random sequence using a computerized random number table with a 1:1 allocation ratio for the control group and ARP group. Allocation concealment was implemented using the sealed opaque envelope method: the random allocation results were sealed in sequentially numbered, opaque envelopes by the statistician, and the envelopes were only opened by the attending nurse after the patient had met all inclusion criteria, provided written informed consent, and completed all preoperative examinations before tooth extraction surgery. No deviations from the random sequence occurred during the study.

#### 2.1.1 Sample size justification

This study did not perform an *a priori* sample size calculation due to the exploratory nature of the combined hard/soft tissue aesthetic assessment for ARP at the study initiation. A *post-hoc* power analysis was subsequently conducted to verify the statistical validity of the sample size, with the primary outcome defined as the change in alveolar bone width at 12 months (the most clinically relevant index for alveolar ridge preservation in aesthetic zones (11, 15). Based on previous meta-analyses (1, 8) reporting a mean difference (MD) of 0.8–1.0 mm in alveolar bone width loss between ARP and conventional extraction groups, with a pooled standard deviation (SD) of 0.4 mm, we used G*Power 3.1 software to calculate the statistical power. With a sample size of 30 patients per group, an alpha level of 0.05 (two-tailed), and an expected MD of 0.6 mm (the minimal clinically important difference for alveolar bone width in implant dentistry (17), the *post-hoc* power for the primary outcome was 98.7%, which exceeds the recommended 80% power for clinical trials. This confirms that the sample size in the present study is sufficient to detect the clinically meaningful differences in the primary outcome between the two groups. For secondary outcomes (alveolar bone height loss, soft tissue aesthetic scores), the *post-hoc* power was also >90%, further supporting the statistical adequacy of the sample size.

This prospective randomized controlled trial was conducted in accordance with the ethical principles outlined in the Declaration of Helsinki. The study protocol and informed consent documents were reviewed and approved by the Medical Ethics Committee of Jinshan Hospital Affiliated to Fudan University (Approval No.: JIEC 2023-S21). All participants were fully informed about the nature, purpose, potential risks, and benefits of the study. Written informed consent was obtained from each patient prior to their enrollment in the study and before any study-related procedures were performed.

### 2.2 Treatment protocols

All surgeries were performed by a single senior surgeon with >15 years of experience in oral implantology, to avoid inter-operator bias. Preoperative preparation: (1) oral hygiene instruction (tooth brushing with Bass method, interdental brushing); (2) 0.12% chlorhexidine rinsing for 3 days preoperatively; (3) local anesthesia with 2% lidocaine + 1:100,000 epinephrine.

Control group: Traditional tooth extraction techniques involve the use of atraumatic forceps in order to minimize trauma to the alveolar bone (18). Normal saline was used as irrigation material for the extraction socket; no operations on membrane covering or bone augmentation were performed. Using a simple interrupted pattern, 4-0 Non-absorbable Surgical Suture (with Needle, Silk Suture) (Fosun Pharma, China) were used to achieve primary closure.

ARP group: The extraction of the teeth was performed in the same non-invasive way as the control group. The granulation tissue and pieces of root were carefully removed from the extraction socket during the debridement process. An operating microscope (manufacturer: Zumax, model: OMS2350 China) was used as an auxiliary instrument during the operation. Implanting a paste made of *β*-tricalcium phosphate (*β*-TCP) biomaterial (0.25 g particles with a diameter of 0.5–1.0 mm, Geistlich, Switzerland) into the socket with the graft material slightly elevated 1 mm above the alveolar crest (to account for physiological resorption of the biomaterial) was performed (19). A 10 × 15 mm collagen barrier membrane from Geistlich Bio-Gide® of Switzerland was cut according to the socket and allowed to hang over the alveolar crest by 2–3 mm. To fix it firmly, 4 corners were stitched using 5-0 Non-absorbable Surgical Suture (with Needle, Silk Suture) (Fosun Pharma, China) to prevent any displacement of membrane. A horizontal mattress suture pattern with 4-0 absorbable was used in the primary closure, keeping in mind to reduce tension on the soft tissue (20).

Postoperative management: (1) antibiotics: Cefixime 200 mg 2 times a day for 3 days (Clindamycin 300 mg for Cefixime allergic cases), (2) analgesics: ibuprofen 200 mg as needed for pain, (3) oral hygiene - 0.12% chlorhexidine-rinses from 3 to 1 times daily for 2 weeks (avoid forceful rinsing due to the risk of detachment of the membranes), (4) food restrictions: no hard/chewy food for 4 weeks (avoid use of extraction side).

### 2.3 Data collection

#### 2.3.1 Radiographic assessment

Preoperative and 6/12 months post extraction CBCT scans were obtained by a Planmeca Finland with the following standard protocol: 110 kV, 9 mA, voxel size 0.125 mm, scan time 18 s. All scans were performed with the patient in a standardized head position (maxillary occlusal plane parallel to the horizontal plane, sagittal plane aligned with the midline of the scanner) to minimize positional variability between different time points. The reconstruction parameters included a field of view (FOV) of 10 × 10 cm (focused on the extraction site), a reconstruction slice thickness of 0.5 mm, and an increment of 0.25 mm. Two independent radiologists with more than 10 years of experience blinded to group allocation analyzed the images using Romexis software (version 5.5.2, QR, Italy). Discrepancies >0.2 mm between the two radiologists were resolved by a third senior radiologist to ensure measurement reliability.

##### 2.3.1.1 Standardized identification of the alveolar crest and 3 mm subcrestal measurement plane

Alveolar crest identification: For all time points (preoperative, 6-month, 12-month), the alveolar crest at the extraction site was defined as the most coronal point of the labial cortical bone at the labial midpoint of the socket, with the mesial and distal adjacent tooth cementoenamel junctions (CEJ) as fixed anatomical reference landmarks. The radiologists marked this point on the axial, coronal, and sagittal CBCT views simultaneously to ensure spatial consistency of crest identification across follow-up time points.

3 mm subcrestal width measurement plane: After identifying the alveolar crest at the labial midpoint, a horizontal reference plane perpendicular to the tooth axis (or adjacent tooth axis for the extracted site) was established at 3 mm apical to the identified crest using the Romexis software's linear measurement and plane reconstruction function. The alveolar ridge width was measured as the horizontal distance between the labial and lingual cortical plates on this standardized plane, consistent with the standard level for implant placement evaluation (21).

##### 2.3.1.2 Measurement parameters and clinical rationale for single-point measurement

All linear measurements were performed exclusively at the labial midpoint of the extraction socket, selected as the primary measurement site for two key clinical reasons: (1) the labial cortical plate at the socket midpoint is the most susceptible to post-extraction resorption (15), especially in the anterior aesthetic zone, and its dimensional changes directly determine soft tissue aesthetic outcomes and implant placement feasibility; (2) the labial midpoint is a consistent and easily reproducible anatomical landmark across CBCT views, minimizing inter-observer variability in measurement site selection.

Alveolar ridge height: The vertical distance from the standardized alveolar crest (labial midpoint) to the deepest point of the residual extraction socket, measured along the tooth axis on the sagittal CBCT view at the labial midpoint.

Alveolar ridge width: The horizontal distance between the labial and lingual cortical plates on the 3 mm subcrestal standardized plane, measured at the labial midpoint on the axial CBCT view (21).

The value before surgery was subtracted from the amount after surgery to get the bone collapse. When there were more than 0.2 mm between the two readings of the radiologists, a third radiologist would be called in to help settle the dispute. Notably, CBCT image fusion and volumetric superimposition were not performed in this study due to the technical specifications of the scanning equipment at the time of study initiation.

#### 2.3.2 Plaster model evaluation

Concurrent CBCT and oral imprints were taken including addition of silicone (Express XT, 3M ESPE, USA) prior to surgery and repeated at 6/12 months. In 24 h, plaster models were poured by type IV dental stone (Fujirock EP, GC, Japan). The following criteria were used by two prosthodontists (with over ten years of experience) to independently assess the soft tissue morphology (1) gingival margin continuity (continuous/disrupted); (2) contour regularity (smooth/mildly irregular/moderately irregular/severely irregular); and (3) keratinized mucosa width (measured with a periodontal probe, with dimensions of 2 mm or more being deemed adequate (21). Discussion resulted in the unification of differences.

### 2.4 Soft tissue aesthetic scoring

A 10-point composite exploratory aesthetic scoring system was developed for this study, with the Pink Esthetic Score (PES) (22) as the theoretical basis. This modified scoring system has not been previously validated in published literature and is defined as an exploratory outcome measure for the multidimensional assessment of soft tissue aesthetics after alveolar ridge preservation (ARP). The total score (range: 4–10 points) was equally divided into two components, with 5 points assigned to soft tissue color and 5 points to soft tissue morphology, respectively. Two independent experts with more than 10 years of clinical experience (an oral implantologist and a prosthodontist) were blinded to the group allocation of patients, and the final score for each patient was the average of the two experts' evaluations to ensure inter-observer objectivity.

#### 2.4.1 Soft tissue color score (5 points)

5 points: A uniform pink color consistent with the adjacent gingiva, with clear vascular patterns (indicating sufficient blood perfusion (23);

3.5 points: Light pink, slightly lighter than adjacent gingiva, with mild local pallor (no obvious vascular reduction);

3 points: Light pink, with scattered pale areas (reduced vascular density);

2.5 points: Markedly pallid color, significantly lighter than adjacent gingiva, without obvious vascular patterns (suggesting poor blood supply).

#### 2.4.2 Soft tissue morphology score (5 points)

5 points: Smooth and continuous gingival margin arc, consistent with the curvature of adjacent teeth; soft tissue fullness matches adjacent sites (no depression or bulging);

3.5 points: Mild gingival margin irregularity (≤1 mm deviation from adjacent arc), basically continuous arc; slight fullness deficiency (≤0.5 mm depression) without clinical aesthetic impairment;

3 points: Moderate gingival margin irregularity (1–2 mm deviation), local depression (0.5–1 mm);

2 Points: obvious gingival margin disruption (≥2 mm deviation), severe contour disorder (≥1 mm depression or bulging), presenting obvious clinical aesthetic defects

The total score was calculated as the sum of color and morphology scores (range: 4–10 points). Scoring referenced CBCT-derived collapse measurements and plaster model morphology to ensure consistency with objective indicators. The correlation between alveolar bone resorption and aesthetic score deduction was established based on published clinical evidence (15, 22, 24): a bone width collapse of ≥1 mm at the extraction site was associated with a ≥ 0.5 point reduction in the soft tissue morphology score, and a bone height collapse of ≥1 mm was associated with a ≥ 0.3 point reduction in the total aesthetic score. This scoring correlation was standardized before the start of the study and strictly applied in the expert evaluation process to ensure the consistency between subjective aesthetic scoring and objective radiographic bone resorption indicators (24).

### 2.5 Statistical analysis

First, the study outcomes were clearly classified as primary and secondary to standardize the statistical analysis: the primary outcome was the change in alveolar bone width at the extraction site at 12 months postoperatively; the secondary outcomes included alveolar bone height loss at 12 months, soft tissue morphology evaluated by plaster models, and soft tissue color/morphology aesthetic scores at 6 and 12 months. All data were analyzed using IBM SPSS Statistics 26.0 (IBM Corp., Armonk, NY, USA). The Shapiro–Wilk test was used to test whether data was normally distributed. The continuous variables, of which age, bone height/width and aesthetic scores were, were indicated as mean ± standard deviation (x ± s). On the other hand, the categorical variables, including gender and gingival margin status were presented in counts and percentages (n, %). We used paired t-tests or repeated measures Analysis of Variance (ANOVA) with Bonferroni *post-hoc* correction consistently applied for multiple comparisons to control for type I error to compare pre- and post-operative groups, and independent t-tests or chi-square tests for categorical variables were used to compare between groups. With the use of Spearman correlation (r_s_) with 95% confidence intervals (95% CI) we studied the correlation between the esthetic scores and the bone collapse. An intraclass correlation coefficient (ICC) of 0.8 or higher was considered to indicate high reliability between observers and 0.6 to 0.8 was considered to show moderate reliability. A level of *α* = 0.05 was used.

## 3 Results

### 3.1 Baseline characteristics

The sixty patients were randomly allocated to the control group (*n* = 30) and to ARP group (*n* = 30). In the twelve months that follows not one patient was lost to follow up. Table 1 summarizes the fact that there were no baseline differences between the two groups (*P* > 0.05). The average age of the control group was 35.8 ± 8.0 years with average age of the ARP group was 36.2 ± 8.3 years (t = 0.38, *P* = 0.704). In the control group, there were 16 men and 14 females and in ARP group, there were 15 males and 15 females (*χ*^2^ = 0.13, *P* = 0.717). The difference of control and ARP groups on pre-extraction alveolar bone height was 6.5 ± 1.1 mm (t = 0.47, *p* = 0.64) and the difference of the bone width was 5.7 ± 0.5 mm (t = 0.12, *p* = 0.90) between the two groups. The control group and the ARP group had a similar distribution of extraction sites: incisors and canines represented 60.0% (18/30) and 63.3% (19/30) of the extraction sites, respectively (*χ*^2^ = 0.08, *P* = 0.776). Aesthetic grading showed good inter-observer reliability (ICC = 0.81) whereas measurements taken from CBCT showed good inter-observer reliability (ICC = 0.92 and 0.89 for height and breadth respectively). All extraction sockets included in the study were Type I (intact buccal/labial cortical plate with 4 intact socket walls), and there was no significant difference in the distribution of anterior/posterior extraction sites between the two groups (*P* > 0.05), ensuring baseline consistency in socket morphology and extraction site.

**Table 1:** Baseline characteristics of the study population.

Group	Sample size (*n*)	Age (years, mean ± SD)	Gender (M/F, *n*)	Extraction site (Anterior/Posterior, *n*)	Socket type (*n*, %)	Preoperative bone height (mm, mean ± SD)	Preoperative bone width (mm, mean ± SD)
Control	30	35.8 ± 8.0	16/14	18/12	Type I (30, 100.0)	6.5 ± 1.1	5.7 ± 0.5
ARP	30	36.2 ± 8.3	15/15	19/11	Type I (30, 100.0)	7.0 ± 1.0	5.8 ± 0.4
Test Statistic	-	t = 0.38, *P* = 0.704	*χ*^2^ = 0.13, *P* = 0.717	*χ*^2^ = 0.08, *P* = 0.776	-	t = 0.47, *P* = 0.64	t = 0.12, *P* = 0.90

SD, standard deviation; M, male; F, female; Anterior teeth = incisors/canines; Posterior teeth = premolars/molars; Socket Type I = intact buccal/lingual cortical plates with 4 intact socket walls (no bony defects); Preoperative bone height/width measured at the labial midpoint (width at 3 mm below the alveolar crest); All groups exclusively included Type I sockets to eliminate confounding from pre-existing bony defects. Statistical significance set at *α* = 0.05.

### 3.2 Alveolar bone volume changes

**Figure 1:**
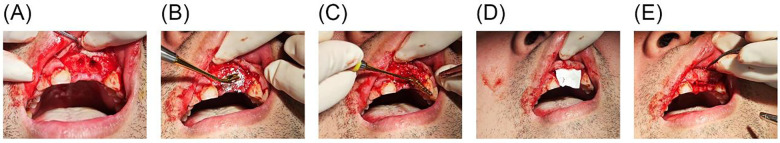
Intraoperative steps of immediate alveolar ridge preservation (ARP) with *β*-TCP biomaterial and collagen barrier membrane. All surgeries were performed via atraumatic tooth extraction technique by a single senior surgeon with >15 years of implantology experience. **(A)** Completed atraumatic tooth extraction with intact Type I extraction socket; **(B)** Thorough debridement of the extraction socket to remove granulation tissue and residual root fragments; **(C)** *β*-TCP biomaterial (0.5–1.0 mm particles, Geistlich, Switzerland) grafting with 1 mm elevation above the alveolar crest (AC) to compensate for physiological resorption; **(D)** Trimming and placement of collagen membrane (Geistlich Bio-Gide®) with 2–3 mm overhang on the alveolar crest, fixed by 5-0 absorbable sutures; **(E)** Soft tissue primary closure with horizontal mattress suture to reduce soft tissue tension. AC, alveolar crest; ES, extraction socket; BP, buccal cortical plate; LP, lingual cortical plate; *β*-TCP, *β*-tricalcium phosphate. Scale bar = 10 mm.

Based on the standardized CBCT radiographic assessment protocol with fixed anatomical landmarks and a single measurement site at the labial midpoint, the following steps were taken during the ARP procedure which follows standardized intraoperative steps (Figure 1): (A) finishing tooth extraction; (B-C) accurate insertion of *β*-TCP biomaterial in the extraction socket to fill the extraction site; (D) covering of the grafted site with collagen barrier membrane in order to safeguard the graft and direct tissue regeneration; and (E) use of a soft tissue relaxation suture to reduce the postoperative tension, which is a key factor in wound healing and stability of the grafts.

The radiographic results showed that the alveolar bone resorption occurred in both groups after 12 months although at 6 and 12 months, the ARP group had significantly less resorption than the control group (*P* < 0.001, Table 2).

**Table 2:** Comparison of radiographic alveolar bone parameters (mm, mean ± SD).

Parameter	Time point	Control group	ARP group	Bone resorption (mm)	*t*-statistic	*P*-value	Bone resorption rate (mm/month)
Alveolar Bone Height	Preoperative	6.5 ± 1.1	7.0 ± 1.0	–	0.47	0.64	–
	6 months	5.4 ± 0.9	6.5 ± 0.8	1.1 ± 0.3	5.72	<0.001	0.18 (Control); 0.08 (ARP)
	12 months	5.2 ± 0.7	6.3 ± 0.6	1.3 ± 0.4	6.87	<0.001	0.03 (Control); 0.03 (ARP)
Alveolar Bone Width	Preoperative	5.7 ± 0.5	5.8 ± 0.4	–	0.12	0.90	–
	6 months	4.7 ± 0.4	5.2 ± 0.3	1.0 ± 0.3	5.59	<0.001	0.17 (Control); 0.09 (ARP)
	12 months	4.0 ± 0.3	4.7 ± 0.2	1.7 ± 0.4	9.33	<0.001	0.06 (Control); 0.05 (ARP)

Alveolar bone height = vertical distance from the alveolar crest (labial midpoint) to the deepest point of the residual socket; Alveolar bone width = horizontal distance between labial/lingual cortical plates at 3 mm below the alveolar crest (implant placement level); Bone resorption = preoperative value - postoperative value; Bone resorption rate = resorption amount/follow-up months; Measurements performed via CBCT (Planmeca Finland) and Romexis software (v5.5.2); Statistical significance set at *α* = 0.05.

At the 6 month mark, the alveolar height of the control group was 1.1 ± 0.3 mm shorter (from 6.5 ± 1.1 to 5.4 ± 0.9 mm) but the alveolar height of the ARP group was 0.5 ± 0.2 mm shorter (from 7.0 ± 1.0 to 6.5 ± 0.8 mm) (t = 5.72, *P* < 0.001). An increase in height loss in the control group was observed to the extent of 1.3 ± 0.4 mm (to a maximum of 5.2 ± 0.7 mm) at 12 months while in the ARP group the height loss increased to the extent 0.7 ± 0.2 mm (to a maximum of 6.3 ± 0.6 mm) (t = 6.87, *P* < 0.001).

During the period of 6 months, the alveolar width of the control group decreased by 1.0 ± 0.3 mm, from 5.7 ± 0.5 to 4.7 ± 0.4 mm, but the alveolar width of the ARP group decreased by 0.6 ± 0.2 mm, from 5.8 ± 0.4 to 5.2 ± 0.3 mm (t = 5.59, *P* < 0.001). At the 12-month mark, the reduction of width for the control group was found to be 1.7 ± 0.4 mm (4.0 ± 0.3 mm), and for the ARP group the reduction was 1.1 ± 0.3 mm (4.7 ± 0.2 mm) (t = 9.33, *P* ≤ 0.001).

**Figure 2:**
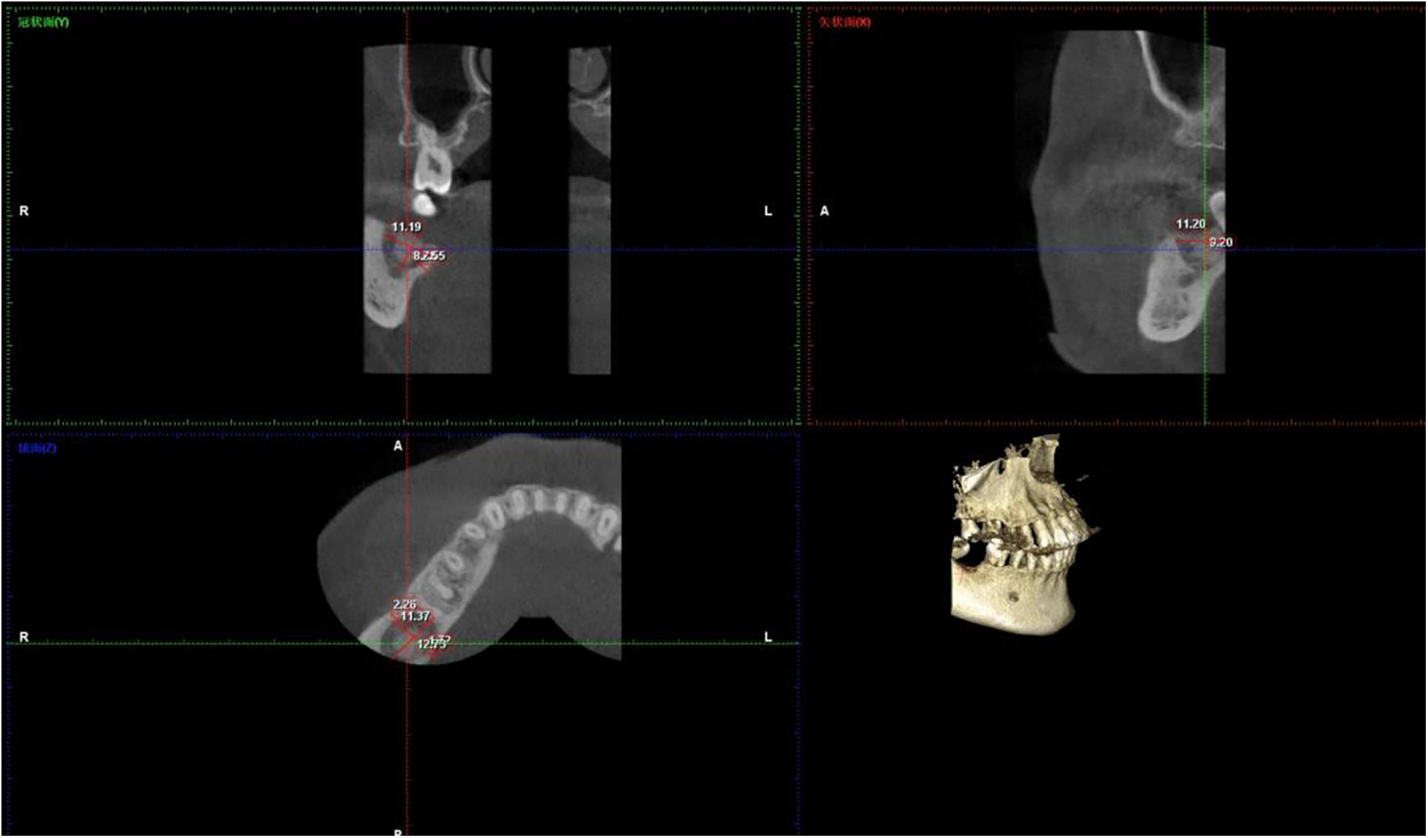
Representative CBCT images of alveolar bone morphology in the control group (conventional tooth extraction) at preoperative, 6 months and 12 months post-extraction. Images show sagittal view (for alveolar bone height measurement) and coronal view (for alveolar bone width measurement) at the labial midpoint of the extraction socket. Alveolar bone height (ABH) was measured as the vertical distance from the alveolar crest (AC) to the deepest point of the residual socket; alveolar bone width (ABW) was measured at 3 mm apical to the alveolar crest (implant placement level). The red line indicates ABH measurement, blue line indicates ABW measurement. Quantitative data are presented as mean ± SD (Table 2), with significant alveolar bone resorption observed at 12 months post-extraction (ABH = 5.2 ± 0.7 mm, ABW = 4.0 ± 0.3 mm). CBCT, cone beam computed tomography; ABH, alveolar bone height; ABW, alveolar bone width; AC, alveolar crest; LCP, labial cortical plate; LgCP, lingual cortical plate; RS, residual socket. Scale bar = 5 mm.

**Figure 3:**
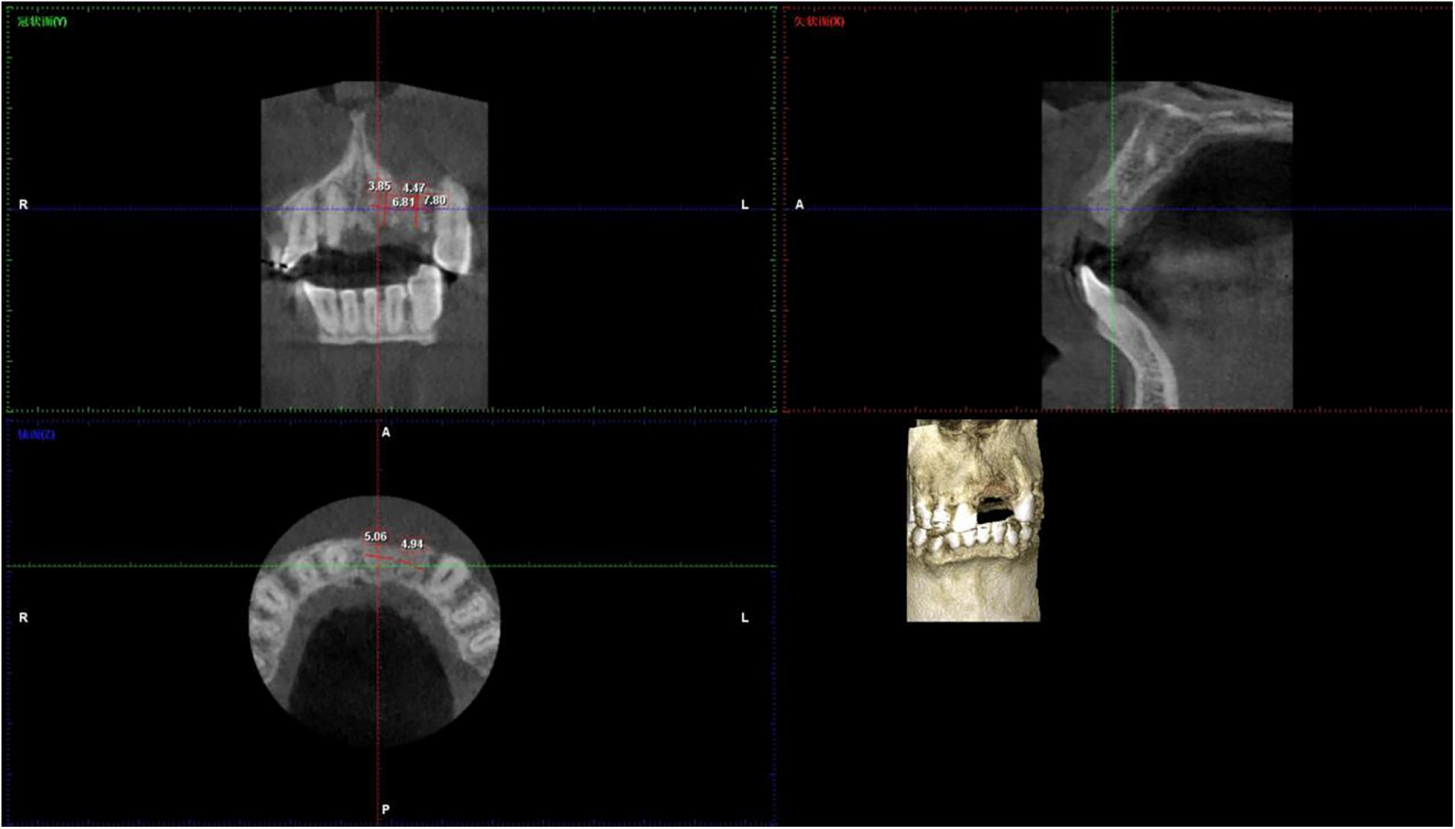
Representative CBCT images of alveolar bone morphology in the ARP group (*β*-TCP + collagen membrane) at preoperative, 6 months and 12 months post-extraction. Images show sagittal view (for alveolar bone height measurement) and coronal view (for alveolar bone width measurement) at the labial midpoint of the extraction socket. Alveolar bone height (ABH) was measured as the vertical distance from the alveolar crest (AC) to the deepest point of the residual socket; alveolar bone width (ABW) was measured at 3 mm apical to the alveolar crest (implant placement level). The red line indicates ABH measurement, blue line indicates ABW measurement. Quantitative data are presented as mean ± SD (Table 2), with significantly less alveolar bone resorption compared with the control group at 12 months post-extraction (ABH = 6.3 ± 0.6 mm, ABW = 4.7 ± 0.2 mm, *P* < 0.001). ARP, alveolar ridge preservation; *β*-TCP, *β*-tricalcium phosphate; CBCT, cone beam computed tomography; ABH, alveolar bone height; ABW, alveolar bone width; AC, alveolar crest; LCP, labial cortical plate; LgCP, lingual cortical plate; RS, residual socket. Scale bar = 5 mm.

The bone resorption rate slowed over time in the control and ARP groups. The rate of change in the former was 0.18 mm/month (0–6 months) and 0.03 mm/month (6–12 months) respectively while it was 0.08 mm/month (0–6 months) and 0.03 mm/month (6–12 months) in the latter. This agrees with the results from previous histological (25) and implies that bone remodeling stabilizes after 6 months. A few CBCT images are shown in Figures 2, 3. These assumptions were founded on the fact that the ARP group experienced less labial bone collapse and a more preserved alveolar ridge contour, than the control group.

### 3.3 Plaster model morphology

At 12 months, the ARP group had significantly better soft tissue morphology than the control group (*χ*^2^ = 53.88, *P* < 0.001, Table 3).

**Table 3:** Soft tissue morphology assessment via plaster models (12 months postoperatively, *n*, %).

Group	*n*	Gingival margin status				Keratinized mucosa width (mm)		t(Inter-group)	*P* (Inter-group)
		Margin disruption^a^ (*n*, %)	Moderate irregularity^b^ (*n*, %)	Mild irregularity^c^ (*n*, %)	Smooth contour^d^ (*n*, %)	Preoperativè	12 months		
Control	30	10 (33.3)	18 (60.0)	2 (6.7)	0 (0.0)	3.2 ± 0.5	2.1 ± 0.4	6.45	< 0.001
ARP	30	0 (0.0)	0 (0.0)	15 (50.0)	15 (50.0)	3.3 ± 0.6	2.8 ± 0.5		
χ^2^	-	53.88	–	–	–	–	–	–	–
P	–	< 0.001	–	–	–	t = 0.68, *P* = 0.50	t = 10.23 (Control); t = 3.87 (ARP)	–	–

^a^ Margin Disruption: ≥2 mm deviation from adjacent gingival arc;

^b^ Moderate Irregularity: 1–2 mm deviation;

^c^ Mild Irregularity: ≤1 mm deviation;

^d^ Smooth Contour: Consistent with adjacent gingival arc; Assessments by two blinded prosthodontists (≥10 years of experience) with excellent inter-observer reliability (ICC = 0.89); Keratinized mucosa width ≥2 mm deemed adequate for implant stability; Intragroup comparisons (preoperative vs 12 months) via paired t-tests.Statistical significance set at *α* = 0.05.

Gingival margin status: No case in the control group had smooth contours; 10 cases (33.3%) had gingival margin disruption, ≥2 mm deviation from adjoining arc, and 18 cases (60.0%) had moderate abnormalities (1–2 mm deviation) and only 2 cases (6.7%) had mild irregularities. Alternatively in the ARP group there were 15 cases of smooth contours and 15 cases of mild irregularities (50.0% of the total), no cases of moderate irregularities or disruption.

Keratinized mucosa width: The width of keratinized mucosa in the control group before the operation was 3.2 ± 0.5 mm and 2.1 ± 0.4 mm after 12 months (t = 10.23, *p* < 0.001), while that of ARP group were 3.3 ± 0.6 mm and 2.8 ± 0.5 mm after 12 months (t = 3.87, *p* < 0.001). A much wider width was shown at 12 months in the ARP group than in the control group (t = 6.45, *P* < 0.001).

Critical parts of stability and cosmetic outcome of implants over a prolonged period including bone volume, soft tissue contour, and keratinized mucosa are retained by ARP according to these studies (26).

### 3.4 Alveolar aesthetic outcomes

Based on the 10-point composite exploratory aesthetic scoring system defined for this study, in both groups, the aesthetic scores of the soft tissues decreased during the entire period of observation, but at 6 and 12 months, the ARP group still had considerably superior values (*P* < 0.001), Table 4).

**Table 4:** Soft tissue aesthetic scores by extraction site (mean ± SD, 10-point scale).

Parameter	Time point	Control group		ARP group		*t/p*(ARP vs. control)	
		Anterior teeth (*n* = 18)	Posterior teeth (*n* = 12)	Anterior teeth (*n* = 19)	Posterior teeth (*n* = 11)	Anterior teeth	Posterior teeth
Soft tissue color score^a^	Preoperative	7.4 ± 0.7	7.2 ± 0.9	7.5 ± 0.6	7.3 ± 0.8	0.42, *P* = 0.676	0.31, *P* = 0.760
	6 months	6.5 ± 0.6	6.7 ± 0.8	7.6 ± 0.5	7.4 ± 0.7	5.79, *P* < 0.001	2.38, *P* = 0.022
	12 months	6.0 ± 0.5	6.2 ± 0.7	7.9 ± 0.4	7.6 ± 0.6	12.15, *P* < 0.001	4.53, *P* < 0.001
Soft tissue Morphology score^b^	Preoperative	7.2 ± 0.8	7.0 ± 1.0	7.4 ± 0.7	7.1 ± 0.9	0.73, *P* = 0.469	0.29, *P* = 0.774
	6 months	6.2 ± 0.7	6.4 ± 0.9	7.3 ± 0.6	7.0 ± 0.8	4.82, *P* < 0.001	1.89, *P* = 0.066
	12 months	5.8 ± 0.6	6.0 ± 0.8	7.8 ± 0.5	7.5 ± 0.7	10.98, *P* < 0.001	3.97, *P* < 0.001
Total aesthetic score^c^	12 months	5.8 ± 0.5	6.3 ± 0.7	8.1 ± 0.4	7.5 ± 0.6	14.32, *P* < 0.001	6.78, *P* < 0.001

^a^ Color Score (max 5 points): 5 = uniform pink with clear vascular patterns; 2.5 = markedly pallid without vascular patterns;

^b^ Morphology Score (max 5 points): 5 = smooth continuous arc with adequate fullness; 2 = disrupted arc with severe contour disorder;

^c^ Total Score = color score + morphology score (range: 4–10 points); Anterior teeth = incisors/canines; Posterior teeth = premolars/molars; Scores averaged by two blinded specialists (implantologist + prosthodontist) with good inter-observer reliability (ICC = 0.81); Statistical significance set at *α* = 0.05.

Color score: There was no effect difference in scores prior to surgery (7.3 ± 0.8 for control and 7.4 ± 0.7 for ARP, t = 0.46, *P* = 0.647). The score of the control group decreased to 6.6 ± 0.7 at 6 months; however, the score of ARP group became 7.5 ± 0.6 (t = 5.47, *P* < 0.001). Control group had their score reduced to 6.1 ± 0.6 at 12 months and ARP group had an increase of 7.8 ± 0.5 (t = 11.25, *P* < 0.001).

Morphology score: There was no significant difference in the scores before surgery (7.1 ± 0.9 for control and 7.3 ± 0.8 for ARP, t = 0.87, *P* = 0.387). On the sixth month, the score for the control group was 6.3 ± 0.8 and for ARP group was 7.2 ± 0.7 (t = 4.36, *P* < 0.001). Scores of 5.9 ± 0.7 and 7.7 ± 0.6, respectively, were obtained at 12 months between the control and the ARP groups (t = 10.17, *P* < 0.001).

Both the total aesthetic score and the relationship between height collapse (r_s_ = −0.78, 95% CI: −0.89 to −0.61, *P* < 0.001) and width collapse (r_s_ = −0.82, 95% CI: −0.91 to −0.68, *P* < 0.001), calculated by Spearman correlation analysis, show that there is a negative relationship between aesthetic scores and bone collapse. Keeping with the “hard tissue support” theory, this proves that preservation of the bone volume is an important factor in the aesthetics of the soft tissues (27). The subgroup analysis of the front teeth showed that the esthetic improvement of ARP was more apparent (12-month total score: 8.1 ± 0.4 vs. control: 5.8 ± 0.5, t = 14.32, *P* < 0.001) than in the back teeth (7.5 ± 0.6 vs. 6.3 ± 0.7, t = 6.78, *P* < 0.001), proving ARP to be clinically useful in the areas where esthetics are important.

## 4 Discussion

Oral rehabilitation is complicated by alveolar bone resorption that results from tooth extraction (28), which impair both the esthetics of the soft tissues and the amount of bone available for implant insertion (29). The results of this study lend credence to the idea that the “active intervention” approach to the management of extraction sockets is effective to prevent bone loss and maintain soft tissue integrity with the use of an immediate ARP with *β*-TCP + collagen membrane.

### 4.1 Mechanisms of ARP in bone and soft tissue preservation

#### 4.1.1 Bone volume preservation

The *β*-TCP biomaterial (Geistlich, Switzerland) used in this study is a synthetic bone substitute with a porous structure (porosity ∼70%), distinct from deproteinized bovine bone mineral (DBBM) products such as Geistlich Bio-Oss®. In ARP, the function of *β*-TCP is double: firstly, as a scaffold, *β*-TCP maintains the three-dimensional space of the extraction socket so that the soft tissue will not grow in, but an osteogenic niche is created (28). Secondly, it has a porous structure (its porosity is around 70%) which promotes the ingrowth of blood vessels, as well as the adhesion of osteoblasts (in fact, histological studies have shown that the formation of new bone inside *β*-TCP scaffolds reaches 30%–40% at 6 months; the biomaterial is gradually resorbed and replaced by native bone (30, 31). Consistent with meta-analysis demonstrating that *β*-TCP decreases bone resorption by 40%–60% when compared to no grafting, the ARP group had 54% and 65% less bone height and width loss at 12 months compared to the control group, respectively (32).

By (1) keeping out connective tissue and epithelial cells from the socket, making sure that osteoblasts are the main cells in the osteogenic microenvironment (33) and (2) keeping moisture and nutrients in, encouraging biomaterial integration (34), the collagen membrane further increases the efficacy of the ARP. Our finding that the ARP group had significantly less bone resorption than the control group is consistent with a previous *in vivo* study that found that collagen membrane covering optimizes new bone formation by 25% compared with no membrane (35).

#### 4.1.2 Soft tissue aesthetic maintenance

One reason the ARP group looks better is the fact that their bone is more densely packed which means there are more attachment sites for soft tissues (gingival fibres and periodontal ligament remnants) and less space for recession in the gums. Another reason is that their preserved three-dimensional bone structure means their vascular networks are hardier, which means soft tissues get enough blood. This is demonstrated by the better colour scores of the ARP group, which indicates improvements in vascularization. Since the labial bone acts as a structural skeleton for the overlying gingival tissue, our correlation research (r_s_=−0.82, 95% CI: −0.91 to −0.68) between the total aesthetic score and the width collapse shows that the preservation of the bone width is especially important for the shape of the soft tissue (15).

At the end of the 12 months, the ARP group had much wider keratinized mucosa than the control group. Because it reduces plaque formation and mechanical damage, keratinized mucosa is very important to implant durability (36). This research explains the fact that ARP works in improving peri-implant health for the long term, which was previously neglected in studies that only examined the short term. It also implies that ARP maintains aesthetics.

### 4.2 Clinical implications of ARP

#### 4.2.1 Aesthetic rehabilitation in anterior teeth

Due to the important visibility of even small gingival recession or irregularity of contour, high esthetic requirements are imposed on anterior teeth (37). It was found in our subgroup analysis that in the ARP group the front teeth, had a 2.3 point higher 12-month aesthetic score than did the control group. This difference was clinically significant, as patients can perceive a difference in the score of ≥1 point (38). As per a prospective research, ARP improves cosmetic success rate of anterior implants (PES (22), by 35% as compared to traditional extraction methods (22). Patients who wish to undergo implant rehabilitation should, therefore, be strongly advised to undergo ARP before extraction of any teeth in the anterior region.

#### 4.2.2 Simplification of implant surgery

Less need for additional bone augmentation is needed when implants are placed in the ARP group because they retain their bone volume. Bone augmentation procedures, such as onlay grafts and sinus lifts, add an additional 60–90 min to the procedure length, a 15% increase to the risk of potential complications (such as infection and graft resorption) and a 30% to 50% increase to the overall cost of treatment (39, 40). Research that we have conducted shows that the ARP group can spare most secondary bone augmentation operations as their 12-month bone height (6.3 ± 0.6 mm) and width (4.7 ± 0.2 mm) are within the minimum standards for routine implant placement (height ≥5 mm, width ≥4 mm) (17, 41). Patients experience greater happiness and a reduced level of stress during treatment as a result of this “one-stage” approach.

#### 4.2.3 Personalized treatment considerations

While ARP is generally an effective approach for alveolar ridge preservation after tooth extraction, it is critical to individualize the procedure based on extraction site heterogeneity (anterior vs. posterior teeth) and socket morphology, as the two sites differ substantially in bone morphology, alveolar socket anatomy, and clinical aesthetic/functional requirements. Anterior and posterior teeth also exhibit distinct post-extraction remodeling patterns, which may affect the efficacy of ARP and subsequent oral rehabilitation outcomes.

##### 4.2.3.1 Anatomical and clinical differences between anterior and posterior extraction sites

Bone morphology and socket anatomy: Anterior teeth (incisors/canines) are located in the maxillary/mandibular anterior alveolar ridge, which is characterized by thin labial cortical bone plates (≤1 mm), a narrow alveolar ridge width, and a shallow, conical alveolar socket with a single root (15, 37). The posterior teeth (premolars/molars) have thicker buccal/lingual cortical bone plates (≥2 mm), a wider and more robust alveolar ridge, and a multi-rooted alveolar socket with a larger volume; the posterior alveolar ridge also has a higher proportion of cancellous bone, which is more prone to horizontal resorption (1, 8).

Aesthetic and functional requirements: Anterior teeth are the core of the facial aesthetic zone, where even minor labial bone resorption (≥0.5 mm) or soft tissue irregularity can lead to visible gingival recession, contour deformity, and aesthetic defects (22, 37). Thus, the primary clinical goal of ARP for anterior teeth is aesthetic preservation of the soft tissue contour and keratinized mucosa width. Posterior teeth are the functional core of mastication, with lower aesthetic requirements; the primary goal of ARP for posterior teeth is volumetric bone preservation to provide sufficient hard tissue support for implant osseointegration and masticatory function (1, 8).

Post-extraction remodeling pattern: The thin labial cortical bone of anterior sites is more prone to vertical resorption after extraction, which directly causes soft tissue recession and aesthetic impairment (15). Posterior sites are more prone to horizontal alveolar ridge resorption due to the high proportion of cancellous bone, which may lead to insufficient bone width for implant placement (1).

##### 4.2.3.2 Clinical implications of site heterogeneity for ARP

The subgroup analysis of this study showed that ARP had a more pronounced aesthetic improvement effect on anterior teeth (12-month total aesthetic score: 8.1 ± 0.4 vs. control: 5.8 ± 0.5, *P* < 0.001) than on posterior teeth (7.5 ± 0.6 vs. 6.3 ± 0.7, *P* < 0.001), which is consistent with the anatomical and clinical characteristics of the two sites. For anterior Type I sockets, ARP with *β*-TCP + collagen membrane can effectively preserve the thin labial cortical bone, maintain the three-dimensional structure of the alveolar ridge, and thus retain the soft tissue anchorage sites and vascular network—this is the key to achieving optimal aesthetic outcomes (15, 37). For posterior Type I sockets, ARP can reduce horizontal bone resorption and avoid secondary bone augmentation procedures, simplifying subsequent implant surgery (17, 41).

For patients with very thin labial bone (<1 mm) in the anterior aesthetic zone, additional soft tissue grafting such as connective tissue graft, to enhance the aesthetic results. Smokers should give up smoking at least three months before ARP, because smoking prevents the process of bone forming and increases the risk of membrane exposure. Additionally, patients with low bone density such as in postmenopausal women might benefit from combining ARP with platelet rich plasma (PRP) or platelet-rich fibrin (PRF) which releases growth factors such as TGF-*β* and PDGF that stimulate osteogenesis (34). To get even better results, further research should be conducted on the combined use of ARP with other bioactive substances.

### 4.3 Limitations and future directions

There are several limitations to this study: (1) No *a priori* sample size calculation was performed at the study initiation, which is a methodological limitation for exploratory clinical studies combining multi-dimensional outcome assessments. However, a detailed *post-hoc* power analysis using G*Power 3.1 confirmed that the sample size (30 patients per group) achieved a statistical power of 98.7% for the primary outcome (alveolar bone width change), with the risk of Type II error reduced to only 1.3%, which ensures the statistical validity of the main study findings. (2) Methodological limitations of CBCT assessment: All alveolar bone measurements were based on linear analysis of two-dimensional CBCT slices with a fixed voxel size, and CBCT image fusion and volumetric superimposition were not performed due to the technical specifications of the scanning equipment at the time of study initiation. In addition, measurements were only performed at the labial midpoint of the extraction socket, which may not fully capture the three-dimensional alveolar ridge remodeling characteristics. Although standardized reference planes and anatomical landmarks were used to minimize positional variability, the alveolar crest itself undergoes vertical remodeling after extraction, which may introduce a small degree of bias to the longitudinal measurement results of bone height and width at the 3 mm subcrestal level. (3) This trial was not registered at [ClinicalTrials.gov](ClinicalTrials.gov), as international clinical trial registration was not a mandatory institutional requirement at the time of study initiation in 2022. All subsequent clinical research by our team will strictly comply with international registration requirements to further improve research transparency. (4) Potential confounding from extraction site heterogeneity: Although this study included both anterior and posterior teeth and performed subgroup analysis to explore the site-specific efficacy of ARP, the two sites were not stratified by sample size during randomization. In addition, only Type I extraction sockets were included, which limits the generalizability of the study results to Type II/III sockets with buccal/labial cortical plate defects. The potential confounding effect of site-related anatomical differences (e.g., bone plate thickness, alveolar ridge width) on post-extraction bone resorption and soft tissue remodeling cannot be completely eliminated, and further stratified randomized controlled trials with site-specific stratification are needed to verify the efficacy of ARP for different extraction sites. (5) The 10-point composite aesthetic scoring system used in this study is an exploratory outcome measure without prior external validation. Although the scoring rules were standardized and correlated with objective radiographic indicators based on published evidence, the results of subjective aesthetic evaluation need to be further verified by a larger sample size and multi-center studies with a validated aesthetic scoring system [e.g., the original PES (22) or modified PES for ARP]. (6) The follow-up period was limited to 12 months, and long-term follow-up (≥3 years) is needed to verify the sustainability of ARP efficacy for implant rehabilitation and long-term soft tissue aesthetic stability. (7) Direct comparisons with other bone graft biomaterials (e.g., bioactive glass, hydroxyapatite) were not performed, and further studies are required to identify the optimal graft material for ARP in different clinical scenarios (e.g., anterior aesthetic zones vs. posterior functional zones). (8) Histological data was not collected; therefore, in future studies, biopsy samples should be used to verify the formation of new bone and the resorption pattern of the graft material in the extraction socket.

Some of the possible areas for future research include: (1) creating customized *β*-TCP scaffolds using 3D printing that is specific to the anatomy of each patient's socket (42); (2) optimizing the rate of osteogenesis (43) by combining ARP with stem cell therapy (i.e., dental pulp stem cells); (3) predicting the success of ARP, using artificial intelligence (44), based on patient characteristics and preoperative CBCT; (4) conducting a multi-center validation study of the exploratory aesthetic scoring system developed in this study to improve its clinical applicability and reproducibility; (5) performing volumetric CBCT analysis and multi-point radiographic measurements to fully capture the three-dimensional alveolar ridge remodeling after ARP; (6) conducting stratified randomized controlled trials with site-specific stratification to verify the efficacy of ARP for different extraction sites and socket types. Both the effectiveness and the personalization of the ARP may be improved through these new developments.

## 5 Conclusion

After tooth extraction, the ARP approach using *β*-tricalcium phosphate (*β*-TCP) biomaterial (Geistlich, Switzerland) in combination with a collagen barrier membrane can significantly reduce alveolar bone resorption. It keeps the width of keratinized mucosa, the continuity of shapes in soft tissues, and achieves good aesthetic results, which especially manifested in the area around the front teeth, which are very sensitive to aesthetic problems. In addition to the reduction in treatment burden, avoidance of most secondary bone augmentation surgeries, and the establishment of a solid hard tissue and cosmetic base for eventual implant rehabilitation, there are several other benefits associated with this approach. However, limitations of the study are the small sample size and short duration of follow-up. To make it even better, future research should use 3D-printed scaffolds in combination with stem cell therapy and large-sample multicenter designs. In the field of individualized oral rehabilitation, generally speaking ARP is considered to be of great therapeutic help.

## Data Availability

The original contributions presented in the study are included in the article/Supplementary Material, further inquiries can be directed to the corresponding author.
